# Electrochemically Switchable Multimode Strong Coupling in Plasmonic Nanocavities

**DOI:** 10.1021/acs.nanolett.3c03814

**Published:** 2024-01-02

**Authors:** Yanji Yang, Rohit Chikkaraddy, Qianqi Lin, Daniel D. A. Clarke, Daniel Wigger, Jeremy J. Baumberg, Ortwin Hess

**Affiliations:** † School of Physics, Trinity College Dublin, Dublin 2, D02 PN40, Ireland; ‡ NanoPhotonics Centre, Cavendish Laboratory, University of Cambridge, J. J. Thomson Avenue, Cambridge, CB3 0HE, U.K.; ¶ School of Physics and Astronomy, University of Birmingham, Birmingham B152TT, England, U.K.; § Hybrid Materials for Optoelectronics Group, Department of Molecules and Materials, MESA+ Institute for Nanotechnology, Molecules Center and Center for Brain-Inspired Nano Systems, Faculty of Science and Technology, University of Twente, 7500AE Enschede, The Netherlands; ∥ CRANN Institute and Advanced Materials and Bioengineering Research Centre, Trinity College Dublin, Dublin 2, D02 PN40, Ireland

**Keywords:** multimode strong coupling, strong coupling control, plasmonic nanocavities, polariton formation

## Abstract

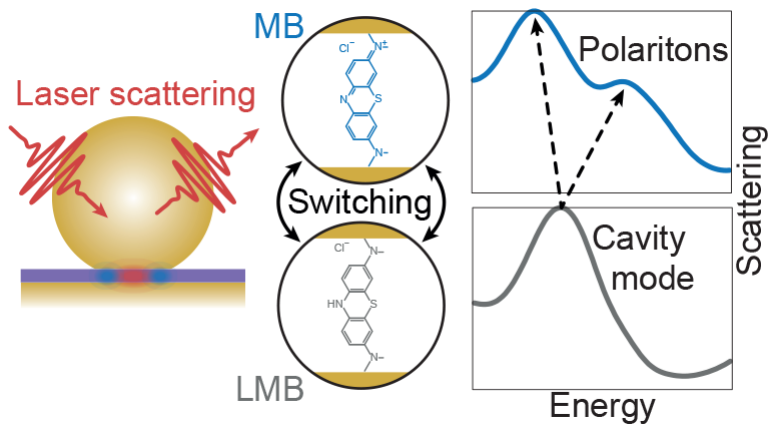

The strong-coupling interaction between quantum emitters and cavities provides the archetypical platform for fundamental quantum electrodynamics. Here we show that methylene blue (MB) molecules interact coherently with subwavelength plasmonic nanocavity modes at room temperature. Experimental results show that the strong coupling can be switched on and off reversibly when MB molecules undergo redox reactions which transform them to leuco-methylene blue molecules. In simulations we demonstrate the strong coupling between the second excited plasmonic cavity mode and resonant emitters. However, we also show that other detuned modes simultaneously couple efficiently to the molecular transitions, creating unusual cascades of mode spectral shifts and polariton formation. This is possible due to the relatively large plasmonic particle size resulting in reduced mode splittings. The results open significant potential for device applications utilizing active control of strong coupling.

The strong coupling regime is central to cavity quantum electrodynamics (cQED), in which the intricate link between quantum emitters and their optical environment leads to the formation of hybrid states that no longer retain their separate identities of light and matter. In this strong coupling regime, the hybrid light-matter states (polaritons) are separated in energy by the vacuum Rabi splitting Ω_*R*_. Since its discovery for many ultracold atoms in 1989^1^ and later for a single atom using a high-finesse optical cavity in 1992,^2^ most investigations of strong coupling have aimed at modifying the optical properties, enabling applications such as photon-based quantum information and low-threshold lasing in semiconductors.^3−6^ Through recent advances in nanofabrication,^7,8^ plasmonic nanocavities with ultralow cavity mode volumes can confine light into nanometre-sized regions far below the diffraction limit. This provides large coupling strengths, , making them a promising approach to access strong coupling at room temperature, at the few- or even singe-molecule level.^9^ While the rapid evolution of nanoplasmonic technologies has enabled a plethora of applications, ranging from single-molecule spectroscopy to solar cells and photothermal cancer treatment,^10−12^ little information on the available polariton modes and how strong coupling can be dynamically controlled in nanoplasmonic environments has been available.

Recently, the selective manipulation of matter by strong coupling and control of interaction strengths from weak to strong coupling has been investigated^13−15^ with the use of tip-enhanced excitation. Active control of the coupling strength has been demonstrated with photoswitchable molecules,^15−19^ polarization-dependent coupling between J-aggregates and gold dimers,^20,21^ and electrostatic gating and thermal tuning of monolayer transition metal dichalcogenides.^22−26^ However, it is challenging to align emitters precisely within a cavity, limiting demonstrations to low yields.^27^

Alternative pathways have emerged that leverage chemistry and material science to guide cQED into new directions and achieve active control of strong coupling easily. In ref (28), Pietron et al. have demonstrated electrochemically controlled switching of strong coupling between molecular vibrational modes and Fabry–Perot cavity modes in the far-infrared spectral range; in particular, the coupling strength was altered through a redox reaction between benzoquinone and dihydroquinone. Naturally, achieving such dynamic switching for electronic transitions interacting with optical-frequency cavity modes would be highly desirable for emerging quantum optoelectronic applications. Our present study reveals that electrochemically triggered redox reactions of methylene blue (MB) molecules can be used to switch on and off strong plasmon-exciton interaction in ultracompact, individual nanocavities featuring 1 nm nanogaps. Our work accesses a regime in which a very limited number of molecules (<100) are highly coupled to the cavity and allow very short switching times compared to bulk approaches with million-fold larger-volume Fabry–Perot cavities. Reversible switching is demonstrated without altering the geometry of the nanocavities. The sequence of simultaneous multimode plasmonic coupling in the system is explored using time-domain simulations, demonstrating the formation of several pairs of polariton states.

The strong coupling is realized using an established model system consisting of a nanoparticle-on-mirror^29−31^ (NPoM) nanocavity hosting a layer of optically active methylene blue (MB) molecules (Figure 1a). The ratio of MB: cucurbit[7]uril (CB[7]) molecules of 1:1 is set by mixing 1 mM solutions of both molecules in sample preparation, to ensure each MB is coupled with one CB[7] (see Supporting Information (SI)). The switching of strong coupling is achieved using an in situ spectro-electrochemical cell.^32^ To theoretically model this nanogap plasmonic NPoM system, we consider the geometry in Figure 1a with dimensions *r* = 40 nm, *w* = 20 nm, and *d* = 1 nm, illuminated by *z*-polarized incident light from the side. The NPoM system is placed in an aqueous medium with a refractive index of 1.33, while the nanogap molecular spacer (purple) has a refractive index of 1.4. As characterized in more detail below, the basic cavity modes^33^ for this geometry arise at 840 nm (1.5 eV, *l*_1_) and 615 nm (2.0 eV, *l*_2_). The gap is scaffolded by a single layer of macrocyclic CB[7] molecules which host the MB molecules and guarantee their vertical alignment in the cavity region.^9,33−35^ This arrangement ensures that the MB molecular dipoles efficiently couple to the dominant plasmonic modes supported by the NPoM. The desired reduction from MB to the leuco-methylene blue (LMB) occurs at −0.5 V vs Ag/AgCl (Figure S2), which switches the optical properties of the active medium, triggered by applying a potential using a standard electrochemical cell (Figure 1a). The redox reaction transforms MB molecules with a transition wavelength of 650 nm (1.9 eV) into LMB, which has an absorption line at 256 nm (4.8 eV)(Figure 1b). This transformation is chromatically characterized by a change from blue to colorless. While MB is close to resonance with the second-order antenna mode *l*_2_ promoting strong coupling, the LMB transition is far detuned from any NPoM mode and as shown below, the coupling is switched off. Ideally, to achieve strong coupling between emitter and cavity, the active plasmonic mode should be tuned into resonance. This is however very challenging for the lowest NPoM mode *l*_1_ because it demands significantly smaller plasmonic particles that render optical cross sections much smaller. With the larger plasmonic particles used here, which bring the *l*_2_ mode into resonance, comes the advantage of smaller mode splittings as discussed below.

**Figure 1:**
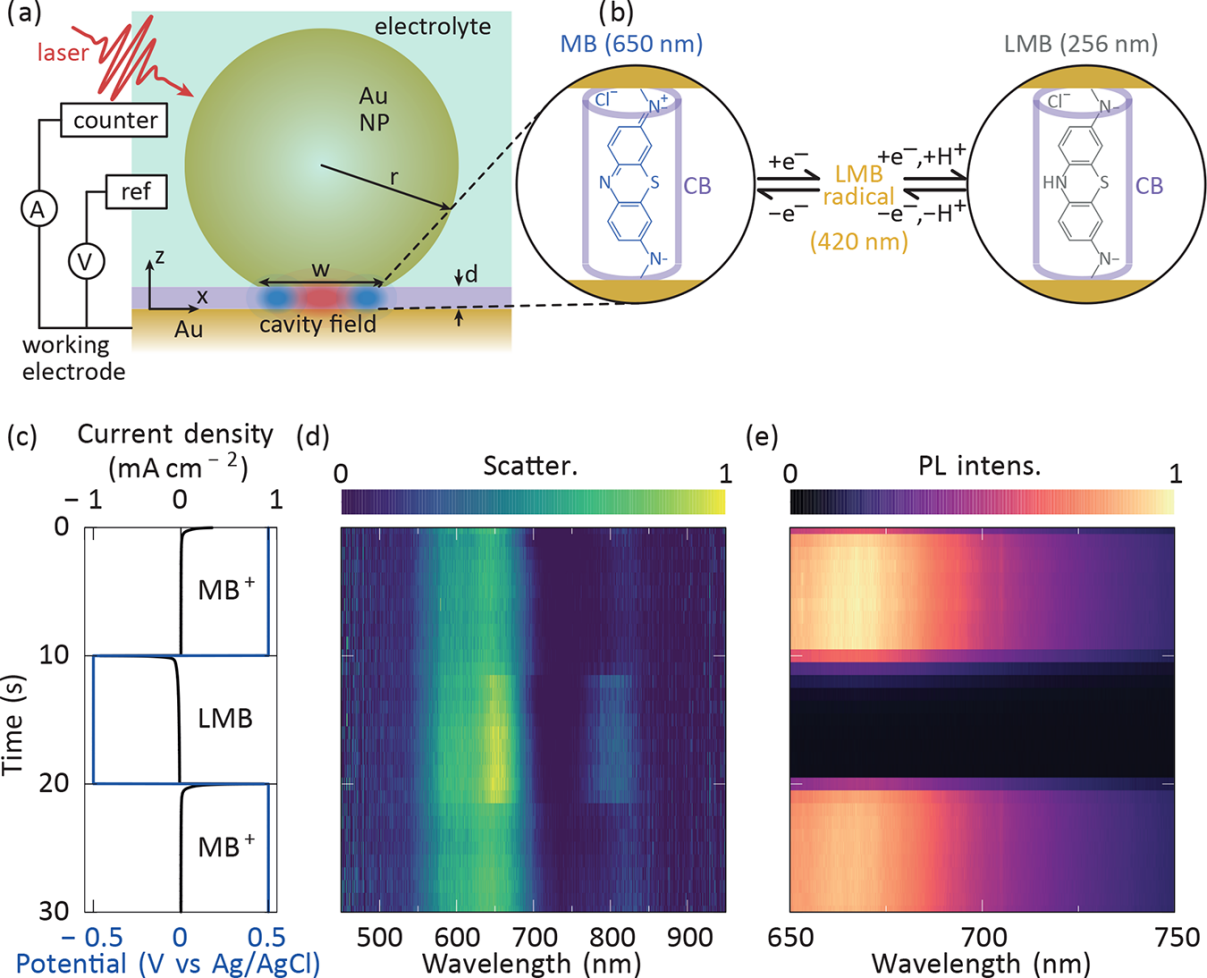
Experimental demonstration. (a) Schematic of the NPoM geometry with cucurbit[7]uril (CB) + methylene blue (MB) molecules in the nanogap layer (purple). The Au nanoparticle has radius *r* = 40 nm and a facet width of *w* = 20 nm, while the nanogap region has a height of *d* = 1 nm and a refractive index of 1.4 (CB). The NPoM is illuminated obliquely at a large angle of incidence. Each CB (purple) molecule hosts a single MB (blue)/leuco-methylene blue (LMB, colorless) molecule ensuring vertical orientation. The circuit is used to perform the reversible redox reactions from MB (transition at 650 nm) to LMB (transition at 256 nm) by adding/removing two electrons. Chronoamperometry scans are between +0.5 and −0.5 V, as determined from cyclic voltammetry (Figure S2). Working, counter and reference electrodes are Au the substrate, Pt mesh, and Ag/AgCl (3 M KCl), respectively. The supporting electrolyte is 0.1 M phosphate buffer solution (PBS) to maintain constant pH 7 during redox. (b) Applied potential (blue) and current density (black) over 30 s, as switch. (c) Evolution of the scattering spectra during the redox reaction time interval in (b). (d) Evolution of the photoluminescence (PL) spectra during the same time interval.

In the experiment, automated tracking microscopy is used to detect dark-field scattering spectra^36^ of hundreds of individual NPoM constructs across the sample surface, where regions of sparsely distributed NPoMs can be selected to ensure that only one construct is within the collection area (see Figure S3). In addition, we perform real-time spectro-electrochemical measurements of the photoluminescence (PL) yield by applying an electrical bias to the gold substrate while irradiating an individual NPoM with continuous-wave laser light at 532 nm. The current density and potential over a time interval of 30 s are plotted in Figure 1c, where the potential is switched between +0.5 V and −0.5 V (vs Ag/AgCl) every 10 s. This triggers the redox conversion of MB to LMB and back to MB as indicated. The reaction is electrochemically controlled by transferring electrons from the working electrode to the reactant in the nanogap over <1 nm distances (Figure 1b). The simultaneous scattering and PL spectra over the same time interval (Figure 1d, e) clearly identify that both signals change when the molecules are switched. From *t* = 10–20 s, the scattering spectrum of LMB is brighter than MB, while its PL spectrum is dark, because it shifts to an entirely different spectral region and is not excited.

The unusual situation here is that strong coupling is set up not with the fundamental mode but with the higher-order mode *l*_2_. This provides an intriguing opportunity to study the additional interplay with further-detuned modes. Since the splitting between plasmonic cavity modes is smaller for larger nanoparticles, *l*_2_ does not dominate the spectrum. To unravel this interplay, we first characterize the NPoM cavity and MB coupling to *l*_2_ in numerical simulations, adding extra damping to the permittivity of the gold, as detailed in the Methods section. The NPoM system in Figure 1a is first simulated without any active medium to determine the optical field profile as a function of the in-plane position x for the 500–900 nm wavelength range of interest (Figure 2a). We find the two dominant plasmonic modes *l*_1_ and *l*_2_ at 840 and 615 nm, respectively (dashed lines). To confirm their mode character the corresponding mode profiles are extracted (Figure 2b). The expected single peak for the lowest energy mode *l*_1_ (green) is seen at 840 nm, while the energetically higher mode *l*_2_ at 615 nm (red) shows two nodes, which stem from a single nodal circle in the *xy* plane (see Figure 3 below). The slight asymmetry observed in the mode profiles of Figures 2b and 3c stems from the choice of incident light direction used in the simulation. The light is incident from the left side of the NPoM geometry with polarization along the *z*-axis into the positive *x*-direction, which breaks the symmetry between *x*- and *y*-directions. While the probing light is incident obliquely in the experiments, our use of laterally incident light in the numerical simulations ensures efficient excitation of the NPoM cavity modes, which bear a largely vertical intracavity field polarization. This choice also ensures the excitation of the *E*_*z*_-polarized lowest *l*_1_ mode.

**Figure 2:**
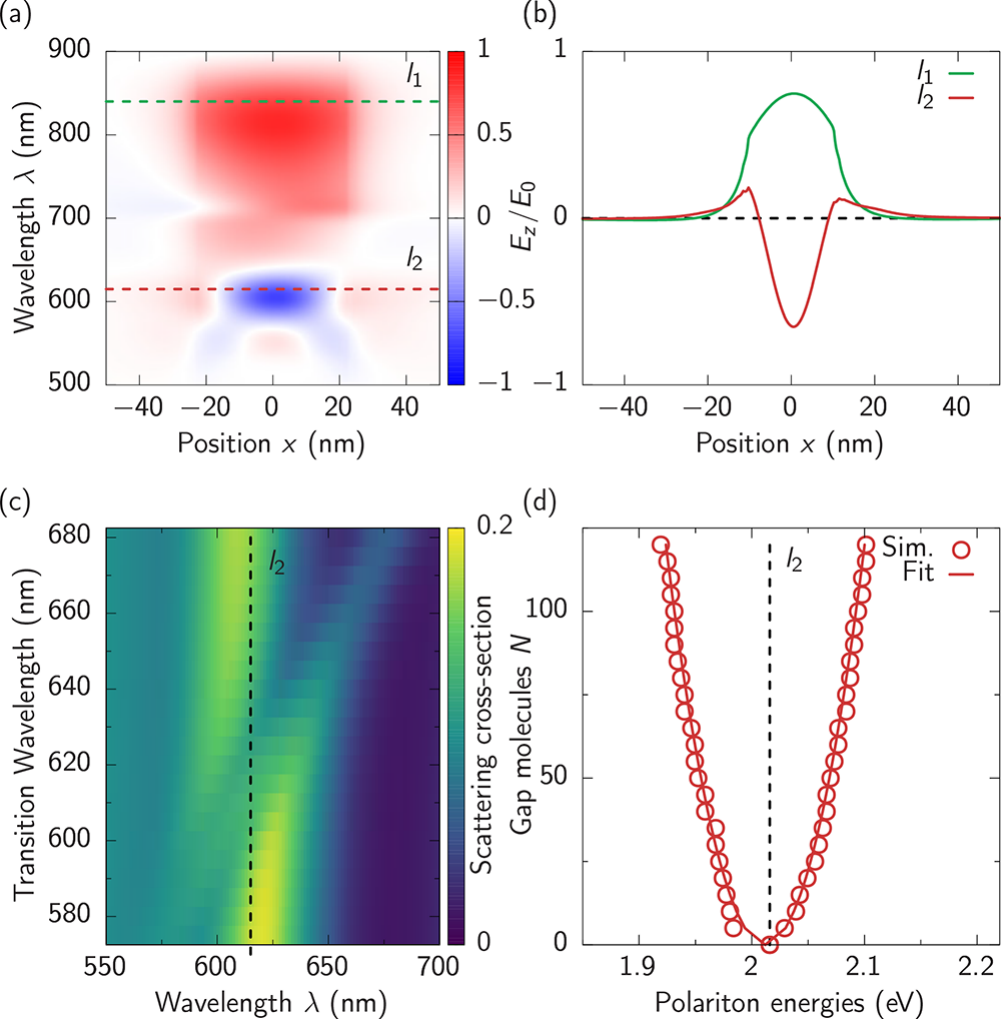
Simulations of the coupled system. (a) Mode profile of the bare NPoM *E*_*z*_ component in the nanogap along the *x*-axis for different wavelengths. (b) *l*_1_ and *l*_2_ mode profiles at the wavelengths marked by dashed lines in (a). (c) Scattering spectra for different emitter transition wavelengths λ_*e*_ tuned across the mode *l*_2_ (dashed line). The anticrossing behavior indicates formation of polariton states. (d) Spectral position of the polaritons (red circles) when the emitters are in resonance with *l*_2_ (dashed line) for an increasing number *N* of molecules in the gap. The splitting between the polaritons follows the fitted  dependence (red solid line).

**Figure 3:**
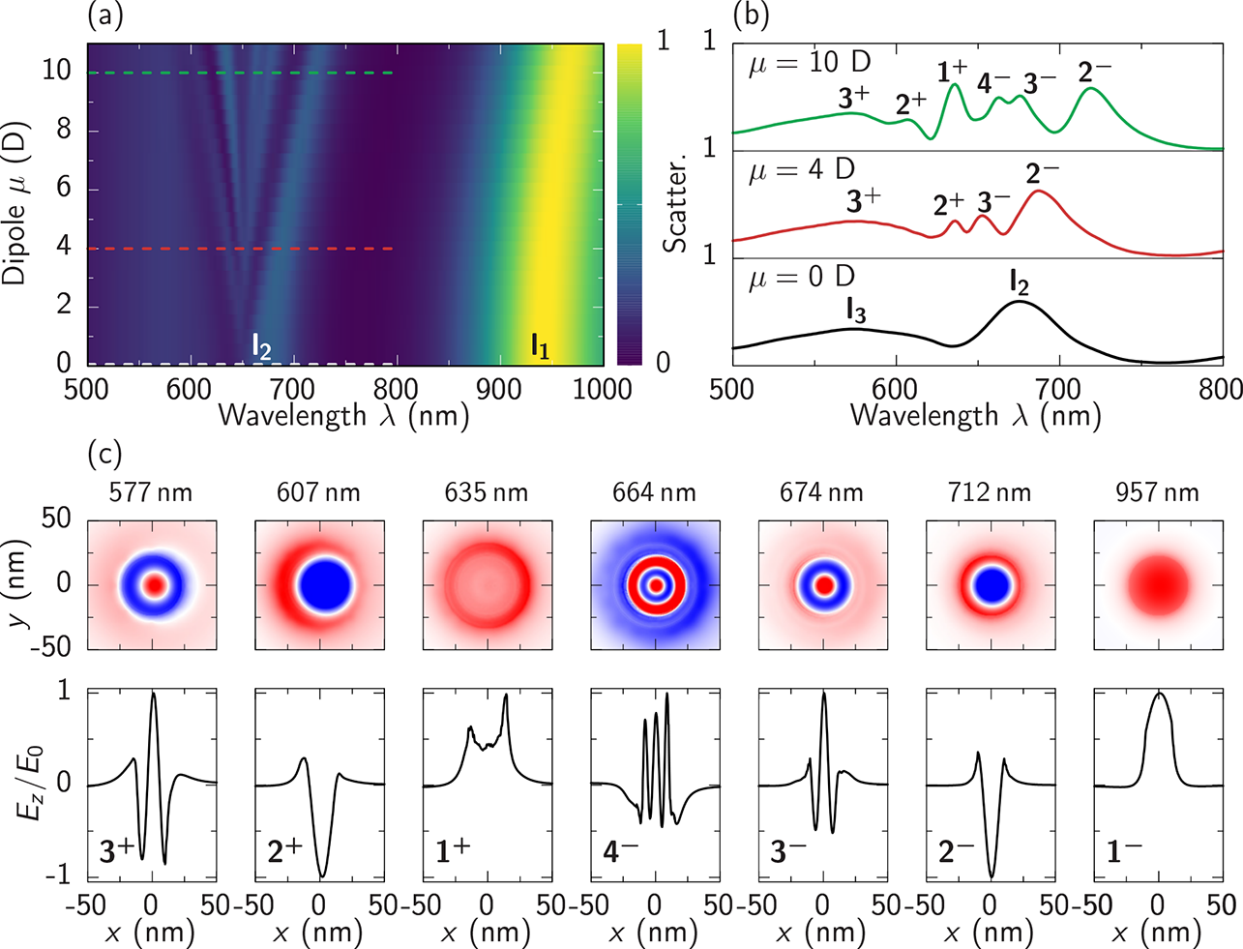
Simulation of multimode strong coupling. (a) Scattering spectra with varying magnitude of dipole moment from μ = 0 to 11 D. (b) Scattering spectra for dipole moments of μ = 0, 4, and 10 D from bottom to top as marked by dashed lines in (a). (c) *E*_*z*_ mode profiles of each peak in the scattering spectrum at μ = 10 D at indicated wavelengths. Top row: field in *xy* plane across the middle of the nanogap (red corresponding to positive, blue to negative *E*_*z*_). Bottom row: 1 D cuts of *E*_*z*_ along the *x*-axis at *y* = 0 nm. Pairs of polaritons can be identified by the number of nodes and are indicated by index 1–4; +/– signs indicate the upper and lower polaritons, respectively.

Since the absorption line of the MB molecules is affected by their dielectric environment, its exact resonance position is not possible to extract from nanogap experiments. Therefore, we model the MB molecules as two-level quantum emitters in our simulations and vary the detuning between the emitters and the cavity mode *l*_2_ by tuning the transition wavelength of the emitters λ_*e*_ from 570 to 680 nm across the *l*_2_ resonance at 615 nm. We choose the density of emitters such that there are on average 50 emitters under the NPoM facet, each with a dipole moment of μ = 4 D and dephasing rate of 5 × 10^13^ rad/s (HWHM Γ of 33 meV). The corresponding results for the scattering spectra are shown in Figure 2c. The clear anticrossing behavior forming two polariton branches around vanishing detuning at λ_*e*_ = 615 nm indicates the occurrence of strong coupling.

From basic models^33^ of multiemitter strong coupling, it is known that the coupling strength scales as  for *N* emitters in resonance with a single plasmonic mode. We conduct a systematic study to investigate the impact of the number of emitters on the coupling strength in the resonant case, since it is difficult to determine the average number of MB molecules in each NPoM cavity in experiment. Scattering spectra are simulated for a range of emitter densities from 0.01 to 0.3 molecules/nm^3^, corresponding to average molecule numbers under the facet from 10 to 120 which is a reasonable range for the experimental molecular density under typical NPoM facets. The polariton peak positions (Figure 2d, red circles) extracted for each emitter density show the peak splitting exhibits the expected square root dependence on the number of emitters (red solid line).

Larger NPoM systems (*r* > 30 nm) have sufficiently dense plasmonic modes that many NPoM modes have relatively small detunings to the MB transition.^37^ Consequently, the MB molecules can couple to multiple plasmonic modes simultaneously and multimode strong coupling emerges for sufficiently large coupling strengths. Surprisingly, we find that the polaritons arise independently from each plasmonic mode, manifesting in the formation of multiple pairs of polaritons observed as peaks in the scattering spectrum.

To systematically study multimode coupling, we simulate the system when varying the dipole moment of emitters from 0 to 11 D while fixing the MB transition at 650 nm with 50 molecules under the NPoM facet. To make evident the multimode coupling behavior in simulations, the extra damping of gold is back to normal and the dephasing rate of emitters is decreased to 1 × 10^13^ rad/s, i.e. HWHM Γ = 6.6 meV. The calculated scattering spectra (Figure 3a) show that coupling between each plasmonic mode and the emitters always exists and is proportional to the dipole moment independent of detuning. Although the effective coupling is significantly reduced for strongly detuned plasmonic modes, the splitting effect from polariton formation leads to energy shifts for all modes. As these shifts overcome the line broadening for sufficiently large coupling, they can be distinguished individually. The scattering spectra for three different dipole moments (Figure 3b, μ = 0, 4, 10 D), marked with dashed lines in Figure 3a, identify several peaks for larger dipole moments. By μ = 10 D, 7 peaks (including the perturbed *l*_1_) are identified, while the shift of polariton peaks between 600–700 nm (Figure 3a) is near-linear with dipole moment, as expected.

More careful analysis identifies pairs of polaritons by comparing the spatial distributions of optical fields at all peaks in the scattering spectrum. At μ = 10 D, spatial distributions of *E*_*z*_(*x*, *y*) through the middle of the nanogap (upper row, Figure 3c) and of *E*_*z*_(*x*) at *y* = 0 nm (bottom row) are extracted for the seven labeled peaks in the scattering spectrum. Each NPoM mode couples to the emitters with a corresponding coupling strength and forms a lower polariton (−) and an upper polariton (+), with these polariton pairs indexed 1–3 (see labels). At μ = 0 D these pairs are degenerate at the respective bare plasmonic modes *l*_1–3_. Extracting the mode positions shows their unusual hierarchy of splitting around the electronic resonance (see SIFigures S5 and S7).

Taking the strong coupling between mode *l*_1_ and the emitters as an example, the hybrid states 1^+^ and 1^–^ are formed at spectral positions 635 and 957 nm. We can pair these peaks through their spatial distributions of *E*_*z*_, as both have similar structures with a single maximum (red circular shape) and no nodes (Figure 3c). This characteristic spatial distribution is inherited from mode *l*_1_ which shows a single antinode in the mode profile. The same procedure can be carried out for the next higher modes leading to the full polariton labeling (Figure 3b, c). The mode profiles become clearer and more pronounced with larger coupling strength. We emphasize how the polariton mode spatial profiles in the multimode strong coupling situation (Figure 3) become nontrivial superpositions of light and matter. We see that the polariton splittings increase with different rates depending on the effective coupling strengths. This means that, for very large dipole moments, different polariton resonances can spectrally intersect, form new hybrid polaritons, and develop additional anticrossings. This is demonstrated in SIFigure S7 depicting a larger dipole moment scan for the parameters from Figure 3a.

We now return to the measured scattering spectra with MB and LMB molecules and compare them with our theoretical predications for coupled and uncoupled nanocavity systems. Our goal is to identify spectral features demonstrating the switching of strong coupling in the system. Comparing the scattering spectra of uncoupled nanocavities in simulation and experiment (Figure 4a, d) shows that the spectral position of the mode *l*_2_ is well matched at ∼615 nm, although the broadening is significantly stronger in experiment. We model the same NPoM system with 50 emitters under the facet, a dipole moment of 4 D, transition wavelength of 650 nm, and total dephasing rate of 1 × 10^14^ rad/s (Γ = 66 meV). For the emitter-coupled nanocavities (Figure 4b, e), a shoulder appears on the high-wavelength side of the *l*_2_ peak, arising from the MB resonance. To quantify all relevant spectral positions, we fit the spectra carefully with Gaussian lineshapes (dashed lines), with the two closest individual peaks plotted (solid curves, Figures 4b, e). Two dependencies are noted: (i) an additional peak appears around 650 nm from the MB resonance, and (ii) both peaks are shifted away from their uncoupled position (dashed vertical lines). Feature (ii) demonstrates the creation of polariton states and the appearance of a near-strong-coupling regime. The splitting of the polariton peaks in the experimental data (Figure 4e) is harder to identify from the scattering spectra collected in an electrochemical cell as our experiments in glass cells with water restrict high-angle light collection. We thus also perform experiments outside the electrochemical cell for 50 different NPoMs (Figure S1). These indeed show a clear difference with and without coupling between the MB molecules and the *l*_2_ plasmon mode. It is important to note that we not only observe a change at the *l*_2_ mode but also a spectral shift of the *l*_1_ mode, as correctly predicted by our simulations. Further we clarify that while Figure 4e does not quite resolve clear strong coupling, in correspondence with our simulations modeling the scattering spectra, we find two polariton peaks at the wavelengths predicted by simulation. We also note in Figure S1 that the different nanoparticles are neither identical in diameter nor identically shaped, which makes perfect agreement between simulations and experiment unviable. However, our simulations predict the correct physical behavior assuming reasonable system parameters.

**Figure 4:**
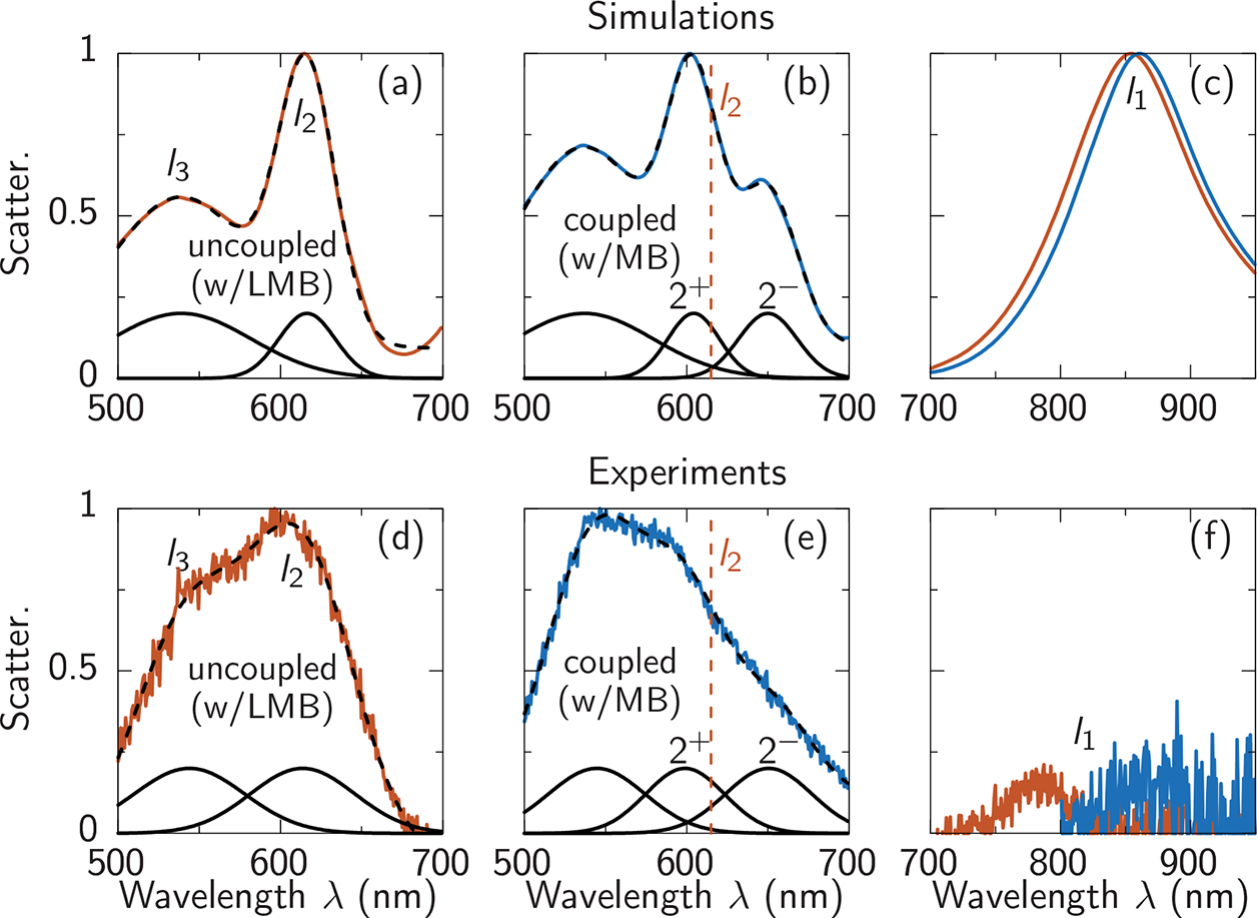
Comparison between scattering spectra in simulation and experiment. (a–c) Simulation and (d–f) experiment, for modes around (a, b, d, e) *l*_2_ and (c, f) *l*_1_, where gap molecules are (a,d) LMB (uncoupled, orange) or (b, e) MB (coupled, blue). The dashed black lines indicate Gaussian fits, while solid black lines indicate the component peaks. The spectral position of *l*_2_ (dashed vertical lines) is indicated in (b, e). The polariton peaks 2^±^ have a combined width of 133 meV in simulation and (181 ± 5) meV in experiment and a splitting of 145 meV in simulation and (172 ± 5) meV in experiment.

Examining now the modes around *l*_1_ (Figure 4c, f) there is only a small shift from the uncoupled (orange) to the coupled (blue) mode in the simulation. This is because the dipole moment of MB molecules (4 D) provides insufficient coupling strength for realizing strong coupling with *l*_1_ in the presence of significant detuning. The larger shift of *l*_1_ in experiment (Figure 4f) suggests additional effects from the redox process, such as changing the refractive index around the nanoparticle.^38,39^ We note the experimental scattering is also broader and weaker than theory predicts, suggesting effects from disorder and/or inhomogeneity.

Strong coupling has been observed in plasmonic nanocavities at room temperature and several experiments have proposed to investigate tuning of the coupling strength. Photoisomerization can also switch electronic resonances, but is typically slow (on time scales of tens to hundreds of minutes). By contrast, our observations show electrochemical control of strong coupling on subsecond time scales. Because the redox process requires electron transfer over subnanometre length scales, our scheme can potentially reach or even exceed GHz operation rates. Redox reactions of MB molecules allow the reversible and nondestructive, on–off switching of the strong coupling without altering the geometry of the nanocavities.

We find that multimode strong coupling in plasmonic systems retains the nanocavity mode symmetry, providing overlapping pairs of polariton modes as the dipole strength increases. This unusual situation comes from the low mode volumes in such plasmonic nanocavities, but also the specific spectrum of their resonance frequencies. Simulations verify that the coupling strength scales as . We anticipate that electrically switchable strong coupling will facilitate many developments in the fields of quantum chemistry, nonlinear optics, and molecular quantum optics.

## Supporting Information Available

(1) Experimental methods: sample design, sample preparation, Spectro-electrochemical cells, Optical measurements, Cyclic voltammetry (CV), NPoM density, MB absorption spectrum; (2) Theoretical methods: FDTD method, Two-level Maxwell-Bloch model, Metal–insulator–metal waveguide model, classical coupled-oscillator model, higher-order hybrid states. (PDF)

## References

[ref1] Raizen M.; Thompson R.; Brecha R.; Kimble H.; Carmichael H. Normal-mode splitting and linewidth averaging for two-state atoms in an optical cavity. Phys. Rev. Lett. 1989, 63, 240 10.1103/PhysRevLett.63.240. 10041018

[ref2] Thompson R.; Rempe G.; Kimble H. Observation of normal-mode splitting for an atom in an optical cavity. Phys. Rev. Lett. 1992, 68, 1132 10.1103/PhysRevLett.68.1132. 10046088

[ref3] Van Loock P.; Munro W.; Nemoto K.; Spiller T.; Ladd T.; Braunstein S. L.; Milburn G. Hybrid quantum computation in quantum optics. Phys. Rev. A 2008, 78, 022303 10.1103/PhysRevA.78.022303.

[ref4] Reithmaier J. P.; Sęk G.; Löffler A.; Hofmann C.; Kuhn S.; Reitzenstein S.; Keldysh L.; Kulakovskii V.; Reinecke T.; Forchel A. Strong coupling in a single quantum dot–semiconductor microcavity system. Nature 2004, 432, 197–200. 10.1038/nature02969. 15538362

[ref5] Christopoulos S.; Von Högersthal G. B. H.; Grundy A.; Lagoudakis P.; Kavokin A.; Baumberg J.; Christmann G.; Butté R.; Feltin E.; Carlin J.-F.; et al. Room-temperature polariton lasing in semiconductor microcavities. Phys. Rev. Lett. 2007, 98, 126405 10.1103/PhysRevLett.98.126405. 17501142

[ref6] Lagoudakis P. G.; Martin M.; Baumberg J. J.; Malpuech G.; Kavokin A. Coexistence of low threshold lasing and strong coupling in microcavities. J. Appl. Phys. 2004, 95, 2487–2489. 10.1063/1.1643191.

[ref7] Anger P.; Bharadwaj P.; Novotny L. Enhancement and quenching of single-molecule fluorescence. Phys. Rev. Lett. 2006, 96, 113002 10.1103/PhysRevLett.96.113002. 16605818

[ref8] Savage K. J.; Hawkeye M. M.; Esteban R.; Borisov A. G.; Aizpurua J.; Baumberg J. J. Revealing the quantum regime in tunnelling plasmonics. Nature 2012, 491, 574–577. 10.1038/nature11653. 23135399

[ref9] Chikkaraddy R.; De Nijs B.; Benz F.; Barrow S. J.; Scherman O. A.; Rosta E.; Demetriadou A.; Fox P.; Hess O.; Baumberg J. J. Single-molecule strong coupling at room temperature in plasmonic nanocavities. Nature 2016, 535, 127–130. 10.1038/nature17974. 27296227 PMC4947385

[ref10] Ferry V. E.; Munday J. N.; Atwater H. A. Design considerations for plasmonic photovoltaics. Adv. Mater. 2010, 22, 4794–4808. 10.1002/adma.201000488. 20814916

[ref11] Wang J.; Boriskina S. V.; Wang H.; Reinhard B. M. Illuminating epidermal growth factor receptor densities on filopodia through plasmon coupling. ACS Nano 2011, 5, 6619–6628. 10.1021/nn202055b. 21761914 PMC3204364

[ref12] Rejiya C.; Kumar J.; Raji V.; Vibin M.; Abraham A. Laser immunotherapy with gold nanorods causes selective killing of tumour cells. Pharmacol. Res. 2012, 65, 261–269. 10.1016/j.phrs.2011.10.005. 22115972

[ref13] Garcia-Vidal F. J.; Ciuti C.; Ebbesen T. W. Manipulating matter by strong coupling to vacuum fields. Science 2021, 373, eabd0336 10.1126/science.abd0336. 34244383

[ref14] Park K.-D.; May M. A.; Leng H.; Wang J.; Kropp J. A.; Gougousi T.; Pelton M.; Raschke M. B. Tip-enhanced strong coupling spectroscopy, imaging, and control of a single quantum emitter. Sci. Adv. 2019, 5, eaav5931 10.1126/sciadv.aav5931. 31309142 PMC6625822

[ref15] Berrier A.; Cools R.; Arnold C.; Offermans P.; Crego-Calama M.; Brongersma S. H.; Gomez-Rivas J. Active control of the strong coupling regime between porphyrin excitons and surface plasmon polaritons. ACS Nano 2011, 5, 6226–6232. 10.1021/nn201077r. 21776964

[ref16] Schwartz T.; Hutchison J. A.; Genet C.; Ebbesen T. W. Reversible switching of ultrastrong light-molecule coupling. Phys. Rev. Lett. 2011, 106, 196405 10.1103/PhysRevLett.106.196405. 21668181

[ref17] Moilanen A. J.; Hakala T. K.; Törmä P. Active control of surface plasmon–emitter strong coupling. ACS Photonics 2018, 5, 54–64. 10.1021/acsphotonics.7b00655.

[ref18] Baudrion A.-L.; Perron A.; Veltri A.; Bouhelier A.; Adam P.-M.; Bachelot R. Reversible strong coupling in silver nanoparticle arrays using photochromic molecules. Nano Lett. 2013, 13, 282–286. 10.1021/nl3040948. 23249360

[ref19] Lin L.; Wang M.; Wei X.; Peng X.; Xie C.; Zheng Y. Photoswitchable Rabi splitting in hybrid plasmon–waveguide modes. Nano Lett. 2016, 16, 7655–7663. 10.1021/acs.nanolett.6b03702. 27960522

[ref20] Schlather A. E.; Large N.; Urban A. S.; Nordlander P.; Halas N. J. Near-field mediated plexcitonic coupling and giant Rabi splitting in individual metallic dimers. Nano Lett. 2013, 13, 3281–3286. 10.1021/nl4014887. 23746061

[ref21] Zhang K.; Chen T.-Y.; Shi W.-B.; Li C.-Y.; Fan R.-H.; Wang Q.-J.; Peng R.-W.; Wang M. Polarization-dependent strong coupling between surface plasmon polaritons and excitons in an organic-dye-doped nanostructure. Opt. Lett. 2017, 42, 2834–2837. 10.1364/OL.42.002834. 28708181

[ref22] Wen J.; Wang H.; Wang W.; Deng Z.; Zhuang C.; Zhang Y.; Liu F.; She J.; Chen J.; Chen H.; et al. Room-temperature strong light–matter interaction with active control in single plasmonic nanorod coupled with two-dimensional atomic crystals. Nano Lett. 2017, 17, 4689–4697. 10.1021/acs.nanolett.7b01344. 28665614

[ref23] Lee B.; Liu W.; Naylor C. H.; Park J.; Malek S. C.; Berger J. S.; Johnson A. C.; Agarwal R. Electrical tuning of exciton–plasmon polariton coupling in monolayer MoS_2_ integrated with plasmonic nanoantenna lattice. Nano Lett. 2017, 17, 4541–4547. 10.1021/acs.nanolett.7b02245. 28613887

[ref24] Li B.; Zu S.; Zhou J.; Jiang Q.; Du B.; Shan H.; Luo Y.; Liu Z.; Zhu X.; Fang Z. Single-nanoparticle plasmonic electro-optic modulator based on MoS_2_ monolayers. ACS Nano 2017, 11, 9720–9727. 10.1021/acsnano.7b05479. 28863263

[ref25] Cuadra J.; Baranov D. G.; Wersall M.; Verre R.; Antosiewicz T. J.; Shegai T. Observation of tunable charged exciton polaritons in hybrid monolayer WS_2_-plasmonic nanoantenna system. Nano Lett. 2018, 18, 1777–1785. 10.1021/acs.nanolett.7b04965. 29369640

[ref26] Kleemann M.-E.; Chikkaraddy R.; Alexeev E. M.; Kos D.; Carnegie C.; Deacon W.; De Pury A. C.; Große C.; De Nijs B.; Mertens J.; et al. Strong-coupling of WSe_2_ in ultra-compact plasmonic nanocavities at room temperature. Nat. Commun. 2017, 8, 1296 10.1038/s41467-017-01398-3. 29101317 PMC5670138

[ref27] Fernández-Domínguez A. I.; Bozhevolnyi S. I.; Mortensen N. A. Plasmon-enhanced generation of nonclassical light. ACS Photonics 2018, 5, 3447–3451. 10.1021/acsphotonics.8b00852.

[ref28] Pietron J.; Fears K.; Owrutsky J.; Simpkins B. Electrochemical modulation of strong vibration–cavity coupling. ACS Photonics 2020, 7, 165–173. 10.1021/acsphotonics.9b01339.

[ref29] Ciracì C.; Hill R.; Mock J.; Urzhumov Y.; Fernández-Domínguez A.; Maier S.; Pendry J.; Chilkoti A.; Smith D. Probing the ultimate limits of plasmonic enhancement. Science 2012, 337, 1072–1074. 10.1126/science.1224823. 22936772 PMC3649871

[ref30] Tserkezis C.; Esteban R.; Sigle D. O.; Mertens J.; Herrmann L. O.; Baumberg J. J.; Aizpurua J. Hybridization of plasmonic antenna and cavity modes: Extreme optics of nanoparticle-on-mirror nanogaps. Phys. Rev. A 2015, 92, 053811 10.1103/PhysRevA.92.053811.

[ref31] Kongsuwan N.; Demetriadou A.; Horton M.; Chikkaraddy R.; Baumberg J. J.; Hess O. Plasmonic nanocavity modes: From near-field to far-field radiation. ACS Photonics 2020, 7, 463–471. 10.1021/acsphotonics.9b01445.

[ref32] Wright D.; Lin Q.; Berta D.; Földes T.; Wagner A.; Griffiths J.; Readman C.; Rosta E.; Reisner E.; Baumberg J. J. Mechanistic study of an immobilized molecular electrocatalyst by in situ gap-plasmon-assisted spectro-electrochemistry. Nature Catalysis 2021, 4, 157–163. 10.1038/s41929-020-00566-x.

[ref33] Baumberg J. J.; Aizpurua J.; Mikkelsen M. H.; Smith D. R. Extreme nanophotonics from ultrathin metallic gaps. Nat. Mater. 2019, 18, 668–678. 10.1038/s41563-019-0290-y. 30936482

[ref34] Kongsuwan N.; Demetriadou A.; Chikkaraddy R.; Benz F.; Turek V. A.; Keyser U. F.; Baumberg J. J.; Hess O. Suppressed quenching and strong-coupling of purcell-enhanced single-molecule emission in plasmonic nanocavities. ACS Photonics 2018, 5, 186–191. 10.1021/acsphotonics.7b00668.

[ref35] Ojambati O. S.; Chikkaraddy R.; Deacon W. D.; Horton M.; Kos D.; Turek V. A.; Keyser U. F.; Baumberg J. J. Quantum electrodynamics at room temperature coupling a single vibrating molecule with a plasmonic nanocavity. Nat. Commun. 2019, 10, 1049 10.1038/s41467-019-08611-5. 30837456 PMC6400948

[ref36] De Nijs B.; Bowman R. W.; Herrmann L. O.; Benz F.; Barrow S. J.; Mertens J.; Sigle D. O.; Chikkaraddy R.; Eiden A.; Ferrari A.; et al. Unfolding the contents of sub-nm plasmonic gaps using normalising plasmon resonance spectroscopy. Faraday Discuss. 2015, 178, 185–193. 10.1039/C4FD00195H. 25793557

[ref37] Benz F.; Chikkaraddy R.; Salmon A.; Ohadi H.; De Nijs B.; Mertens J.; Carnegie C.; Bowman R. W.; Baumberg J. J. SERS of individual nanoparticles on a mirror: size does matter, but so does shape. J. Phys. Chem. Lett. 2016, 7, 2264–2269. 10.1021/acs.jpclett.6b00986. 27223478 PMC4916483

[ref38] Di Martino G.; Turek V.; Tserkezis C.; Lombardi A.; Kuhn A.; Baumberg J. Plasmonic response and SERS modulation in electrochemical applied potentials. Faraday Discuss. 2017, 205, 537–545. 10.1039/C7FD00130D. 28879365

[ref39] Di Martino G.; Turek V.; Lombardi A.; Szabó I.; de Nijs B.; Kuhn A.; Rosta E.; Baumberg J. Tracking nanoelectrochemistry using individual plasmonic nanocavities. Nano Lett. 2017, 17, 4840–4845. 10.1021/acs.nanolett.7b01676. 28686457

