# Supplementary material for: Electrochemically Switchable Multimode Strong Coupling in Plasmonic Nanocavities
Source: Nano Lett. 2024 Jan 2;24(1):238–44. doi: 10.1021/acs.nanolett.3c03814 (PMC10786147; doi:10.1021/acs.nanolett.3c03814)
Supplement: Supplementary file 1 — nl3c03814_si_001.pdf [file nl3c03814_si_001.pdf]

Electrochemically switchable multimode strong coupling in plasmonic nanocavities

(Supporting Information)

Yanji Yang,[†] Rohit Chikkaraddy,[‡,¶] Qianqi Lin,[‡,§] Daniel D. A. Clarke,[†] Daniel Wigger,[†] Jeremy J. Baumberg,[*,‡] and Ortwin Hess[*,†,∥]

†*School of Physics, Trinity College Dublin, Dublin 2, D02 PN40, Ireland*

‡*NanoPhotonics Centre, Cavendish Laboratory, University of Cambridge, J. J. Thomson Avenue, Cambridge, CB3 0HE, UK*

¶*School of Physics and Astronomy, University of Birmingham, Birmingham B152TT England, UK*

§*Hybrid Materials for Opto-Electronics Group, Department of Molecules and Materials, MESA+ Institute for Nanotechnology, Molecules Center and Center for Brain-Inspired Nano Systems, Faculty of Science and Technology, University of Twente, 7500AE Enschede, the Netherlands*

∥*CRANN Institute and Advanced Materials and Bioengineering Research, Trinity College Dublin, Dublin 2, D02 PN40, Ireland*

E-mail: jjb12@cam.ac.uk; ortwin.hess@tcd.ie

S1 Experimental Methods

Sample design:

Our system is the nanoparticle-on-mirror (NPoM) with an ultrasmall gap of $\sim$ 1 nm and designable properties, filled with a monolayer of spacer cucurbit[n]uril (CB[n]) molecules capable of hosting active quantum emitters in the form of methylene blue (MB) molecules in the nanogap. These macrocyclic CB[n] molecules help to preclude aggregation of the MB molecules and to assemble them with deterministic orientation along the z-direction in the cavity, via supramolecular guest-host chemistry. In particular, CB[7] can accommodate exactly one MB molecule (MB:CB[7] = 1:1) within its hollow, hydrophobic interior, while carbonyl portals at either end of the molecules bind them with their rims flat onto the gold surface. When a monolayer of CB[7] is first deposited on the gold mirror and suitably filled with MB molecules, gold nanoparticles can then bind on top to form the filled nanocavities, with the MB molecules vertically aligned within the nanogap. Previous spectroscopic measurements with empty CB[n] molecules have shown that such a particle-mirror gap is approximately 0.9 nm in extent and presents an effective refractive index of 1.4.[1]

Sample preparation:

1 mM CB[7] and 1 mM MB are mixed in aqueous solution, to ensure the ratio of MB:CB[7] is 1:1.[2] The Au substrate is immersed in this solution, rinsed with water, and dried with nitrogen. The 40 nm citrate capped AuNPs (BBI Solutions) are drop casted onto the Au substrate for 10 s. After this, the substrate is rinsed and dried.

Spectro-electrochemical cells:

The cells are made by 3D printed chambers with adhering glass coverslips using double sided tapes. The supporting electrolyte used is phosphate buffer solution (PBS) composed of 0.1 M monobasic and dibasic potassium phosphates. The NPoM samples are immersed in the electrolyte, and placed under the coverslips. The Au substrate is used as the working electrode. A Ag/AgCl reference electrode (3 M KCl, eDAQ ET072, Green Leaf Scientific) and a Pt mesh counter electrode (Alfa Aeser) are used. A potentiostat (Autolab PGSTAT204,

Metrohm) is used for chrono-amperometry.

Optical measurements:

Photoluminescence spectroscopy is recorded on a modified Olympus BX51 with a 633 nm laser at the power of 100 µW. The spectra are recorded with an Andor camera coupled to a Triax 320 spectrometer.

Dark-field scattering spectroscopy is performed on a modified Olympus BX51 with a halogen lamp. The spectra (lower case 's') are recorded using a fibre-coupled OceanOptics QE65000. Automated tracking microscopy was performed using a Python particle-tracking code.[1] A standard diffuser (Labsphere) is used as a reference to normalise white light scattering.

For both spectroscopy methods, excitation and collection happen through a 0.8-NA Olympus LWD BF/DF objective.

The 50 recorded scattering spectra in Fig. S1 without MB (a, uncoupled) and with the blue-emitting MB molecules in (b) evidence a clear difference with and without strong coupling between the MB molecules and NPoM modes. In addition, slight variations from nanoparticle to nanoparticle show that all cavities are slightly different due the particle sizes and facets, which makes an exact reproduction in simulation impossible.

Cyclic voltammetry (CV):

The reduction potential of MB hosted in CB within the NPoM geometry is measured by a typical CV measurement. As MB reduction at a Au electrode completes at -0.5 V,[3] the potential window is chosen from -0.5 to $+0.5$ V. The recorded CV in Fig. S2 shows reduction of MB completes at -0.5 V, and this value is used for chronoamperometry in Fig. 1 in the main text.

NPoM density:

In Fig. S3 we present an exemplary image of the collection area (a) and NPoMs (b). We find less dense areas where a single NPoM response can be collected.

MB absorption spectrum:

Figure S4 shows an absorption spectrum of an ensemble of MB molecules with a significant

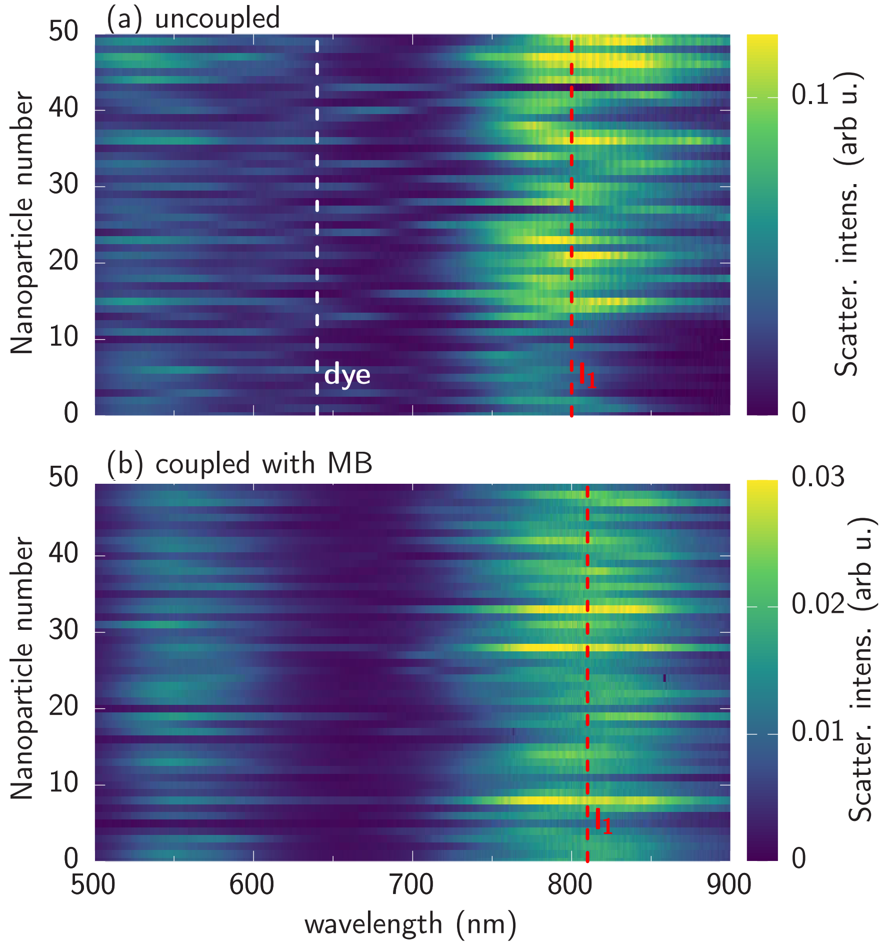

Figure S1: Dark-field scattering spectra from fifty different individual NPoMs outside the electrochemical cell, with and without MB. The white dashed line marks the dye transition wavelength, the red dashed line is l_1.

inhomogeneous broadening of around 57 meV.

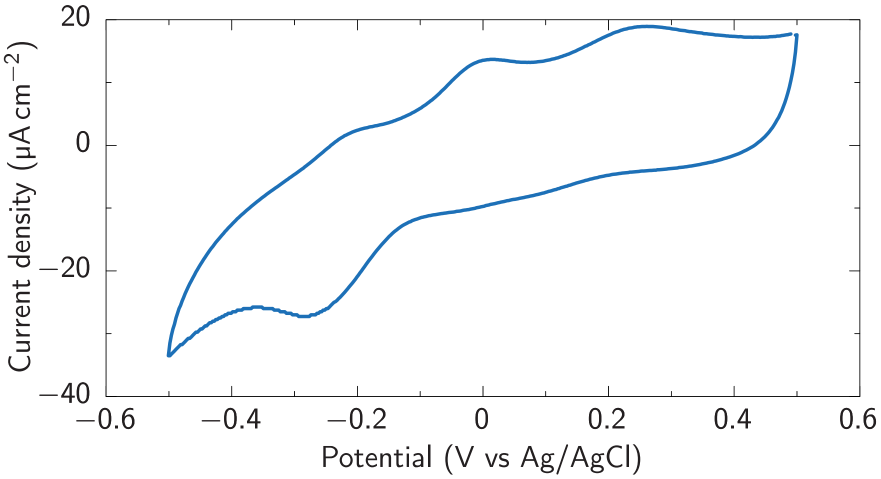

Figure S2: Cyclic voltammetry (CV) of MB:CB[7] NPoMs, confirming reduction potential at -0.5 V. Scan rate $= 100$ mV s^{-1}.

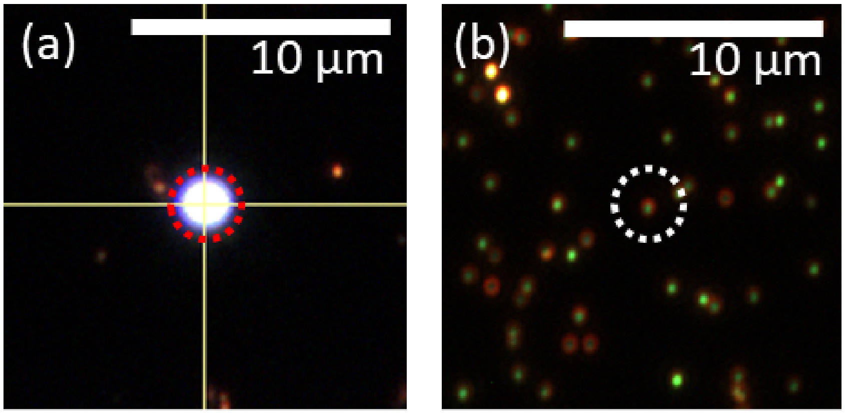

Figure S3: (a) Collection area identified by illumination along the collection path, circled in red for clarity. (b) To ensure only one NPoM is in the collection area (circled white), regions of sparsely distributed NPoMs are selected. NP diameter $= 80$ nm. 100x, 0.8 NA objective.

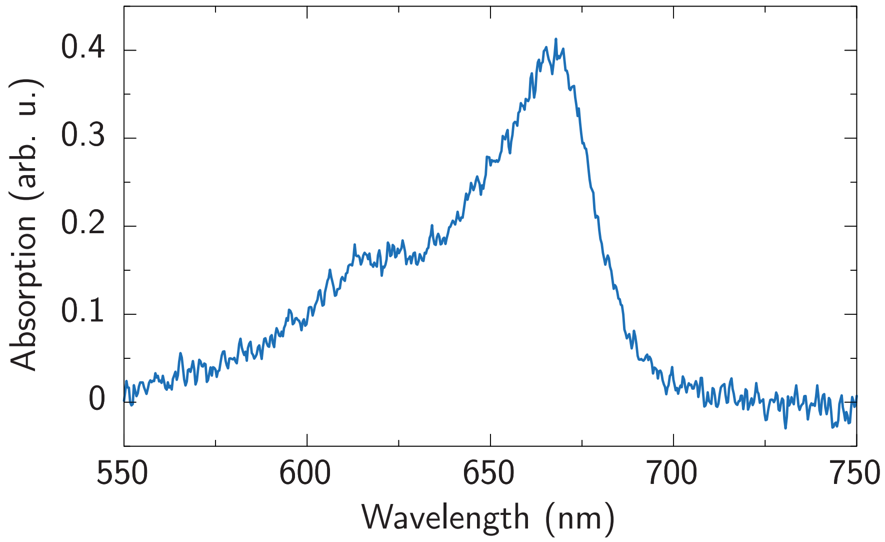

Figure S4: Absorption spectrum of an ensemble of MB molecules with a significant inhomogeneous broadening.

S2 Theoretical Methods

FDTD method:

Our full-dimensionality finite-difference time-domain (FDTD) simulations[4] of the NPoM nanocavity are performed using Ansys Lumerical software (2022 R1.1 release). The gold (Au) nanoparticle (depicted in Fig. 1a of the main text) is modelled as a truncated sphere of diameter $D = 80$ nm, with a facet width of $w = 20$ nm. The CB[7] layer is treated as a uniform dielectric medium with refractive index 1.4[1] and thickness $d = 1$ nm, separating the Au nanoparticle from an infinite Au substrate. For the permittivity of Au, ϵ_{Au}, we employ the data of Johnson and Christy,[5] but to account for additional dissipation mechanisms stemming from inhomogeneities or imperfections of the NPoM constructs in the experiment, we enhance the damping through multiplication of the imaginary part of ϵ_{Au} by 1.2 (as in Fig. 2 and Fig. 4). The ambient dielectric environment of the NPoM is assumed to be water with a refractive index of $n = 1.33$. Our FDTD calculations employ a 1 nm mesh size in a region of dimensions $(100 \times 100 \times 100)$ nm^3 containing the NPoM structure, which is progressively refined to a mesh size of 0.25 nm in the nanogap region, ensuring an accurate description of the spatial variations in the plasmonic near-field therein. The injection of an

exciting plane wave into the simulation domain (with angle of incidence $\theta_i = 90°$) and the calculation of the scattering cross-section in a spectral range from 300 nm to 1000 nm are performed using the total-field scattered-field (TFSF) technique;[4] in particular, the total-field region is defined by a cuboidal volume of dimensions $(200 \times 200 \times 180)$ nm^3 containing the NPoM and the scattering cross-section is proportional to the total power transmission across the TFSF boundary. The entire simulation domain is $(1 \times 1 \times 1)$ µm^3, and open boundaries are described by means of perfectly matched layers[4] using standard profiles.

Two-level Maxwell-Bloch model:

In order to study the interaction of the NPoM nanocavity modes with an active layer of MB emitters, we employ a self-consistent, two-level Maxwell-Bloch description[6,7] with Hamiltonian

$$\hat{H} = \hat{H}_e + \hat{V}(t) = \hbar\omega_e \hat{\sigma}^\dagger \hat{\sigma} - \hat{\boldsymbol{\mu}} \cdot \boldsymbol{E}(t). \tag{1}$$

Here, $\hat{H}_e = \hbar\omega_e \hat{\sigma}^\dagger \hat{\sigma}$ is the field-free Hamiltonian of the two-level molecular emitter, in which ω_e denotes the transition frequency, while $\hat{\sigma}^\dagger$ and $\hat{\sigma}$ are the raising and lowering operators, respectively. The operator $\hat{V}(t) = -\hat{\boldsymbol{\mu}} \cdot \boldsymbol{E}(t)$ describes the dipolar interaction between an emitter and the electromagnetic field, in which $\hat{\boldsymbol{\mu}} = \boldsymbol{\mu}(\hat{\sigma}^\dagger + \hat{\sigma})$ is the dipole moment operator and $\boldsymbol{\mu}$ is a dipole matrix element. For our simulations, we assume that the dipole moment is oriented in the z-direction, so that $\boldsymbol{\mu} = \mu \boldsymbol{e}_z$.

We regard each two-level emitter as an open quantum system which, in addition to its dipole interaction with an external field, experiences relaxation and dephasing. The temporal evolution of the emitter density operator $\hat{\rho}$ under these conditions can be described by means of the following quantum master equation in Lindblad form,

$$\frac{\partial \hat{\rho}}{\partial t} = -\frac{i}{\hbar}[\hat{H}, \hat{\rho}] + \frac{\gamma_r}{2}(2\hat{\sigma}\hat{\rho}\hat{\sigma}^\dagger - \hat{\sigma}^\dagger\hat{\sigma}\hat{\rho} - \hat{\rho}\hat{\sigma}^\dagger\hat{\sigma}) + \frac{\gamma_d}{2}(\hat{\sigma}_z\hat{\rho}\hat{\sigma}_z - \hat{\rho}), \tag{2}$$

where $\hat{\sigma}_z = \hat{\sigma}^\dagger\hat{\sigma} - \hat{\sigma}\hat{\sigma}^\dagger$, while γ_r and γ_d are incoherent relaxation and pure dephasing rates, respectively. Forming matrix elements of the left- and right-hand sides of Eq. (2) in a basis

comprising the emitter ground and excited states, we find the following coupled system

$$\frac{\partial \rho_{22}}{\partial t} = -\frac{\partial \rho_{11}}{\partial t} = -\gamma_r \rho_{22} - \frac{2}{\hbar}\boldsymbol{\mu}\cdot\boldsymbol{E}\,\mathrm{Im}(\rho_{12}) \tag{3}$$

$$\frac{\partial \rho_{12}}{\partial t} = \frac{\partial \rho_{21}^*}{\partial t} = -(\Gamma - i\omega_e)\rho_{12} + \frac{i}{\hbar}\boldsymbol{\mu}\cdot\boldsymbol{E}(2\rho_{22} - 1), \tag{4}$$

in which we have introduced the total dephasing rate $\Gamma = \gamma_d + \gamma_r/2$.

For an ensemble of emitters distributed with density N_d, we define the ground- and excited-state population densities as $N_1 = N_d\rho_{11}$ and $N_2 = N_d\rho_{22}$ respectively, and calculate the macroscopic polarization via

$$\boldsymbol{P} = N_d \mathrm{Tr}(\hat{\rho}\hat{\boldsymbol{\mu}}) = 2N_d\boldsymbol{\mu}\mathrm{Re}(\rho_{12}). \tag{5}$$

By separating Eq. (4) according to the real and imaginary parts of the off-diagonal density matrix elements, and using Eq. (5), we can rewrite the coupled system of equations in the form

$$\frac{\partial^2 \boldsymbol{P}}{\partial t^2} + 2\Gamma\frac{\partial \boldsymbol{P}}{\partial t} + (\omega_e^2 + \Gamma^2)\boldsymbol{P} = -\frac{2\omega_e\mu^2}{\hbar}(N_2 - N_1)\boldsymbol{E}(t) \tag{6}$$

$$\frac{\partial N_2}{\partial t} = -\frac{\partial N_1}{\partial t} = -\gamma_r N_2 + \frac{1}{\hbar\omega_e}\left(\frac{\partial \boldsymbol{P}}{\partial t} + \Gamma\boldsymbol{P}\right)\cdot\boldsymbol{E}(t). \tag{7}$$

In this work, we solve numerically MaxwellâĂŹs equations with the optical Bloch equations [Eqs. (6) and (7)] in a completely self-consistent fashion, where the local electric field (computed using the FDTD method) drives a polarization response from the quantum emitters (determined by solving the optical Bloch equations), which in turn couples back to the field. The entire CB[7]:MB layer is regarded as the active medium, with a nominal, single-emitter transition wavelength of $\lambda_e = 2\pi c/\omega_e = 650$ nm (where c is the speed of light in vacuum), a dipole moment of $\mu = 4$ D, a total dephasing rate $\Gamma = 5 \times 10^{13}$ rad/s and an emitter density of $N_d = 1.5 \times 10^{26}/\mathrm{m}^3$, corresponding to $N \approx 50$ MB molecules under the nanoparticle facet.

Metal-insulator-metal waveguide model:

To develop a minimal model that captures the evolution of the polariton pairs for different cavity modes, we invoke the metal-insulator-metal (MIM) waveguide analysis from Ref.[8,9] Specifically, we treat the NPoM nanogap as a one-dimensional waveguide structure comprising an insulator of thickness d and dielectric permittivity $\epsilon_g = n_g^2$, confined between two metal plates with common permittivity ϵ_m. The dispersion of the MIM modes in such a structure is given by[10]

$$\left(\frac{k}{k_0}\right)^2 = n_{\text{eff}}^2 = \epsilon_g + \frac{\gamma}{2}\left[1 + \sqrt{1 + \frac{4[\epsilon_g - \epsilon_m(\omega)]}{\gamma}}\right], \qquad \text{with} \quad n_{\text{eff}} = \frac{\text{Re}(k)}{k_0}, \tag{8}$$

where $k_0 = 2\pi/\lambda$ is the vacuum wavenumber and $\gamma = \{(2\epsilon_g)/(k_0 d \epsilon_m(\omega)]\}^2$. We use this effective refractive index to match the Fabry-Pérot boundary condition for transverse magnetic resonances in a finite-size cylindrical gap of height d, which leads to the problem of self-consistently solving the equation[11]

$$\alpha_{mn} - \beta_{mn} = \frac{\pi d n_{\text{eff}}(\lambda_{mn})}{\lambda_{mn}}, \tag{9}$$

in which α_{mn} is the nth root of the mth order Bessel function J_m and β_{mn} is a mode-dependent phase shift. The latter can be used to account for differences in the frequencies of the confined modes stemming from the more complex geometry of our NPoM system.[12,13]

To mimic the resonant behaviour of the active material in the nanogap (i.e., the spectrally sharp emitters), we adopt the following model permittivity,

$$\epsilon_g(\omega) = \epsilon_B + \frac{f}{\left(\frac{\omega - \omega_0}{\Gamma}\right) + i}, \tag{10}$$

where $\epsilon_B = 1.4^2$ is the background dielectric constant and f is an overall coupling factor, while ω_0 and Γ are the energy and linewidth of the two-level medium, respectively.

This method is of course inadequate to match the exact spectral positions of the polari-

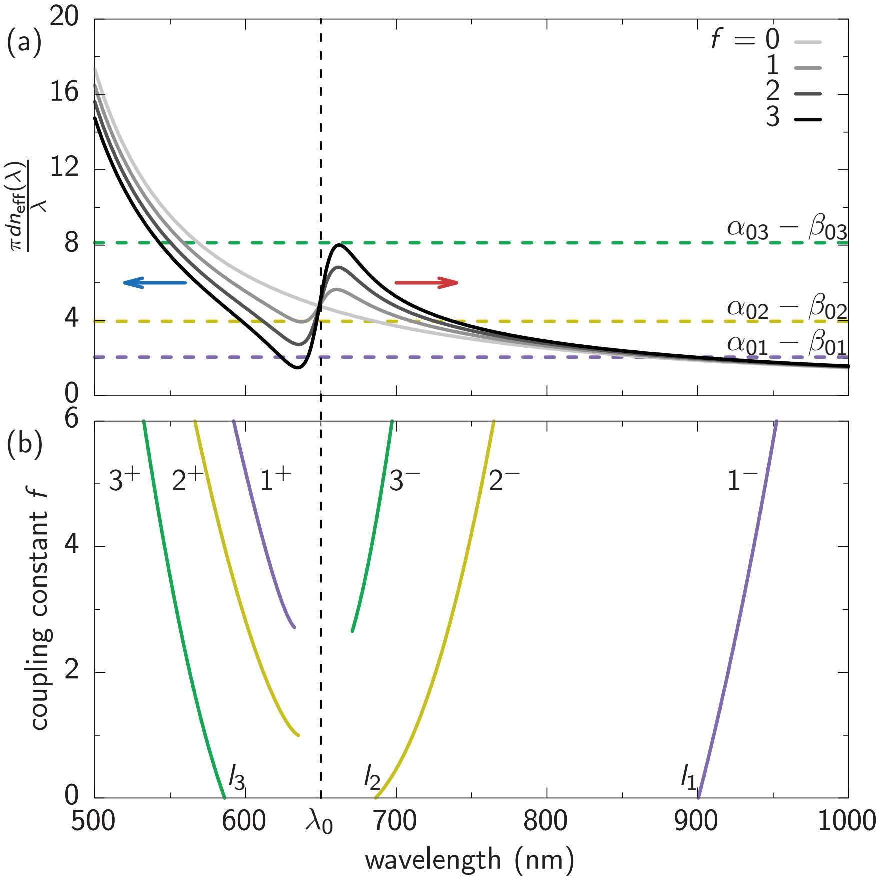

Figure S5: (a) Examples of how the polariton wavelengths are determined. The black/grey curves represent the dispersion in Eq. (9) and the dashed horizontal lines the constants $\alpha_{mn} - \beta_{mn}$. (b) Polariton wavelengths for multimode strong coupling, according to the MIM model, as the coupling constant f increases from 0 to 6. The black dashed line indicates the emitter transition wavelength, while the colored lines show how the polariton positions evolve with increasing strength of interaction between the emitters and the plasmonic modes l_1 (purple), l_2 (yellow) and l_3 (green).

tons, and the coupling strengths between the various plasmonic modes and the emitters are, in general, different. As such, using only a single coupling constant f for all plasmonic modes is insufficient, but it nevertheless suffices to explore qualitative trends in multimode strong coupling, as demonstrated in Fig. S5. The quantities $\alpha_{0n} - \beta_{0n}$ for the plasmonic modes l_n, where $n = 1, 2$, and 3, are shown by the dashed lines in Fig. S5a, and the black/grey curves show the dispersion $\frac{\pi d n_{\text{eff}}(\lambda_{nm})}{\lambda_{mn}}$ for $0 \leq f \leq 3$. As mentioned before, the constant β_{0n} is introduced in the literature to account for mode-dependent boundary behaviour and is used here to adjust the mode frequencies according to our FDTD simulation results. The three points of intersection between the dispersion function with $f = 0$ and the dashed lines [i.e., the solutions of Eq. (9)] determine the spectral positions of the three plasmonic modes l_n in our NPoM system, while they correspond to the polariton peak locations for non-zero f. As shown in Fig. S5b, with increasing coupling constant f the points of intersection to the left of $\lambda_0 = 2\pi c/\omega_0$ are blue-shifting, while those to the right are red-shifting. If the emitter transition is assumed spectrally sharp without any damping, we obtain a unique solution $\lambda_0 = 650$ nm, which is the nominal transition wavelength. However, for non-vanishing broadening as considered here, we take the outermost points of intersection to mark the polariton peak locations, designated n^+ and n^-.

The polariton spectral positions are extracted for coupling constants in the range $0 \leq f \leq 6$, as shown in Fig. S5b. The colored solid lines indicate how the polariton wavelengths change with increasing strength of interaction between the emitters and each cavity mode l_1 (purple), l_2 (yellow) and l_3 (green). The splitting for each pair of polaritons becomes approximately linear and is qualitatively similar to the trends discussed in connection with Fig. 3 of the main text.

Classical coupled-oscillators model:

Simplifying our analysis one step further and neglecting the multimode character of the coupling, we can employ a classical, coupled-oscillators model for each pair of emitter transition and NPoM cavity mode. In this elementary model, we consider a 2×2 Hamiltonian matrix

to describe the coupling between a plasmonic mode l_n and emitters (ω_0) with potentially different dissipation rates κ_n, Γ and frequencies $\omega_a^{(n)}$, ω_0,

$$\hat{H}_{loss} = \begin{pmatrix} \omega_0 - i\Gamma & g_n \\ g_n & \omega_{\mathrm{a}}^{(n)} - i\kappa_n \end{pmatrix}. \tag{11}$$

Since the coupling strength is proportional to the dipole moment, we can write $g_n = g_\mu^{(n)} \cdot \mu$ where $g_\mu^{(n)}$ is the coupling strength per unit dipole moment and assumed to be a constant for each plasmonic mode and a fixed geometry. By diagonalisation, we obtain two eigenvectors of this Hamiltonian corresponding to a pair of polaritons,

$$\omega_\pm = \frac{\omega_0 + \omega_{\mathrm{a}}^{(n)}}{2} - \frac{i}{2}(\Gamma + \kappa_n) \pm \sqrt{\left[\mu g_\mu^{(n)}\right]^2 + \frac{1}{4}\left\{\left[\omega_0 - \omega_a^{(n)}\right] - i(\Gamma - \kappa_n)\right\}^2}. \tag{12}$$

For the numerical simulations, we set $\omega_0 = 1.91$ eV and $\Gamma = 6.6$ meV. In order to obtain the spectral position $\omega_a^{(n)}$ and κ_n for plasmonic mode l_n, we employ a classical dipole source positioned at the center of the NPoM nanogap (as in Fig. 3), attributing the radiative decay to the dominantly radiative l_1 and l_2 cavity modes. By fitting with Lorentzian line-shapes, our peak positions for l_1 and l_2 are found to be $\omega_a^{(1)} = 1.34$ eV and $\omega_a^{(2)} = 1.85$ eV, with decay rates $\kappa_1 = 102$ meV and $\kappa_2 = 92$ meV, respectively.

We fit the polariton peak positions from our full numerical simulations in Fig. 3a of the main text using the functions defined by Eq. (12), where the coupling strength per unit dipole moment $g_\mu^{(n)}$ between the emitters and each plasmonic mode l_n is the quantity to be estimated. Note that higher energy modes usually have a broadening effect and overlap with each other, which renders it difficult to extract the peak positions accurately. Therefore, we focus on the coupling between the emitters and the l_1, l_2 modes for our coupled-oscillators treatment. Fig. S6 presents the fits, where black dots indicate the energy of the uncoupled emitters, and the colored dots show the energies of the polaritons extracted from the FDTD simulations, with purple for l_1 and yellow for l_2. The colored solid lines represent fits of

Eq. (12) to the polariton peak positions with the optimised coupling strength per unit dipole moment $g_\mu^{(1)} = 16.1$ meV/D and $g_\mu^{(2)} = 16.3$ meV/D. By comparing with the linewidth, we find that the strong coupling regime for l_1 and l_2, where $g_n > (\kappa_n + \Gamma)/2$, is achieved for $\mu > 3.4$ D and $\mu > 3$ D, respectively.

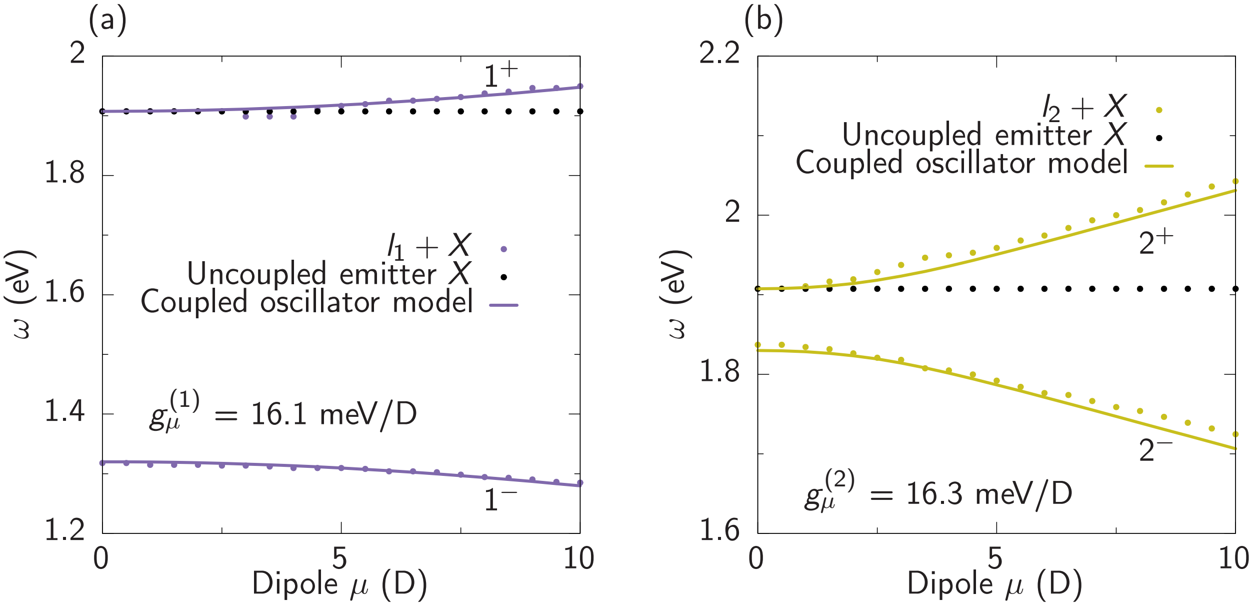

Figure S6: Fitting of the numerical simulation results (colored dots) according to a classical oscillator model (colored solid lines), for coupling between the emitters and (a) the l_1 and (b) the l_2 modes. The black dots indicate the uncoupled emitter energy.

Higher-order hybrid states:

For smaller magnitudes μ of the dipole moment, one can assume that the quantum emitters couple to multiple plasmonic modes simultaneously and independently with different effective coupling strengths, as we discuss in the main text. As the polariton resonances shift with different slopes when increasing μ, a situation can arise in which different polaritons approach the same energy as μ becomes very large. We study this situation in Fig. S7, where we plot the scattering cross-section on a logarithmic scale for better visibility. We clearly see that the polaritons 2^+ and 1^+ approach 3^+ and form anti-crossings with it around $\mu = 15$ D and 20 D, respectively, as marked by the dashed lines. Our numerical results thus point to the formation of higher-order hybrid states from these polaritons.

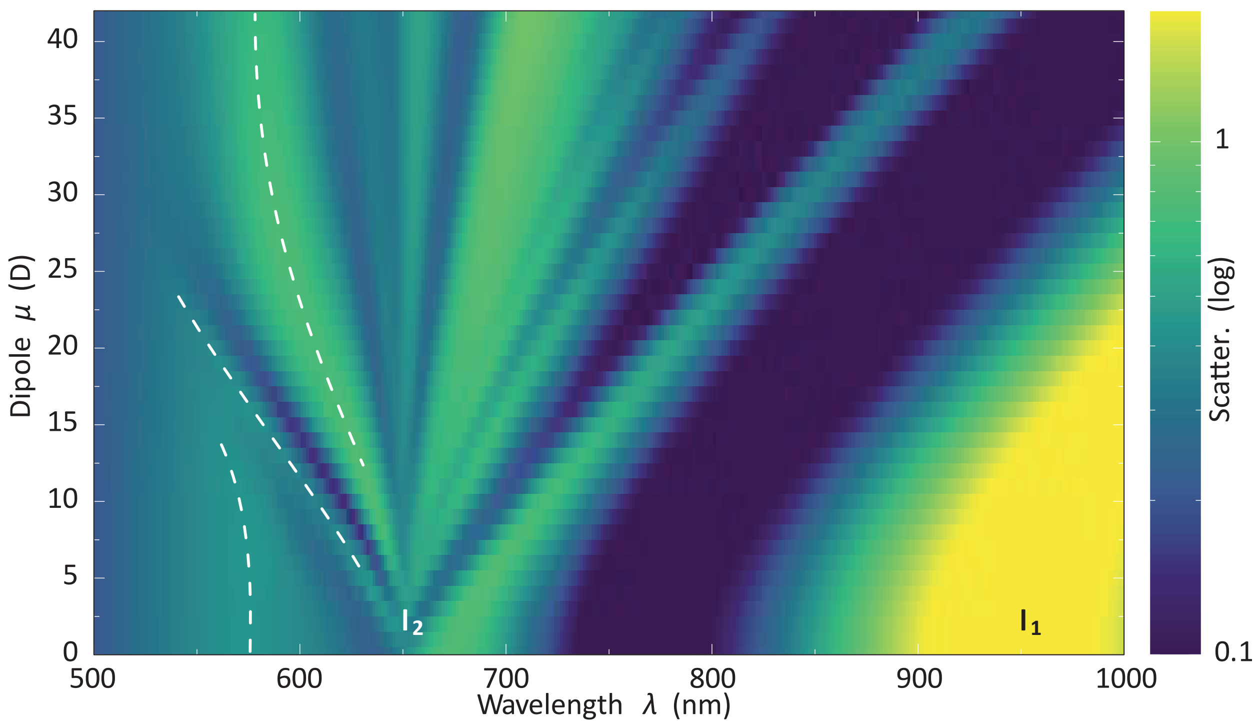

Figure S7: Simulated scattering spectra with varying magnitude of the dipole moment from $\mu = 0$ D to 42 D. The dashed white lines act as a guide to the eye for the polariton resonances.

References

(1) De Nijs, B.; Bowman, R. W.; Herrmann, L. O.; Benz, F.; Barrow, S. J.; Mertens, J.; Sigle, D. O.; Chikkaraddy, R.; Eiden, A.; Ferrari, A. et al. Unfolding the contents of sub-nm plasmonic gaps using normalising plasmon resonance spectroscopy. *Faraday Discuss.* **2015**, *178*, 185–193.

(2) Chikkaraddy, R.; De Nijs, B.; Benz, F.; Barrow, S. J.; Scherman, O. A.; Rosta, E.; Demetriadou, A.; Fox, P.; Hess, O.; Baumberg, J. J. Single-molecule strong coupling at room temperature in plasmonic nanocavities. *Nature* **2016**, *535*, 127–130.

(3) Ahn, W.; Simpkins, B. S. Spectroelectrochemical measurement and modulation of exciton-polaritons. *APL Photonics* **2020**, *5*, 076107.

(4) Taflove, A.; Hagness, S. C. *Computational Electrodynamics: The Finite-Difference Time-Domain Method*; Artech House, Boston, 2005.

(5) Johnson, P. B.; Christy, R. W. Optical constants of the noble metals. *Phys. Rev. B* **1972**, *6*, 4370.

(6) Kongsuwan, N.; Demetriadou, A.; Chikkaraddy, R.; Benz, F.; Turek, V. A.; Keyser, U. F.; Baumberg, J. J.; Hess, O. Suppressed quenching and strong-coupling of purcell-enhanced single-molecule emission in plasmonic nanocavities. *ACS Photonics* **2018**, *5*, 186–191.

(7) Kongsuwan, N.; Xiong, X.; Bai, P.; You, J.-B.; Png, C. E.; Wu, L.; Hess, O. Quantum plasmonic immunoassay sensing. *Nano Lett.* **2019**, *19*, 5853–5861.

(8) Zayats, A. V.; Smolyaninov, I. I.; Maradudin, A. A. Nano-optics of surface plasmon polaritons. *Phys. Rep.* **2005**, *408*, 131–314.

(9) Kuttge, M.; Cai, W.; de Abajo, F. J. G.; Polman, A. Dispersion of metal-insulator-metal plasmon polaritons probed by cathodoluminescence imaging spectroscopy. *Phys. Rev. B* **2009**, *80*, 033409.

(10) Baumberg, J. J.; Aizpurua, J.; Mikkelsen, M. H.; Smith, D. R. Extreme nanophotonics from ultrathin metallic gaps. *Nat. Mater.* **2019**, *18*, 668–678.

(11) Tserkezis, C.; Esteban, R.; Sigle, D. O.; Mertens, J.; Herrmann, L. O.; Baumberg, J. J.; Aizpurua, J. Hybridization of plasmonic antenna and cavity modes: Extreme optics of nanoparticle-on-mirror nanogaps. *Phys. Rev. A* **2015**, *92*, 053811.

(12) Filter, R.; Qi, J.; Rockstuhl, C.; Lederer, F. Circular optical nanoantennas: an analytical theory. *Phys. Rev. B* **2012**, *85*, 125429.

(13) Gordon, R. Light in a subwavelength slit in a metal: Propagation and reflection. *Phys. Rev. B* **2006**, *73*, 153405.
